# Supplementary material for: Impact of sex and marital status on the prevalence of perceived depression in association with food insecurity
Source: PLoS One. 2020 Jun 11;15(6):e0234105. doi: 10.1371/journal.pone.0234105 (PMC7289387; doi:10.1371/journal.pone.0234105)
Supplement: S1 Table — (DOC) [file pone.0234105.s001.doc]

**Supplementary Table 1. A**djusted odds ratio and 95% confidence interval for perceived depression.

|  | **2012, 2013, and 2015** |  |  | **2014** |  |  | **Total (2012-2015)** |  |
| --- | --- | --- | --- | --- | --- | --- | --- | --- |
|  | Adjusted odds ratio  (95% confidence interval) | *P* value |  | Adjusted odds ratio  (95% confidence interval) | *P* value |  | Adjusted odds ratio  (95% confidence interval) | *P* value |
| Sex |  |  |  |  |  |  |  |  |
| Male | 1 |  |  | 1 |  |  | 1 |  |
| Female | 2.31 (1.86-2.88) | < 0.001 |  | 2.79 (1.71-4.56) | < 0.001 |  | 2.39 (2.00-2.92) | < 0.001 |
| Marital status |  |  |  |  |  |  |  |  |
| Never married | 1.33 (1.09-1.63) | 0.01 |  | 1.59 (1.02-2.47) | 0.04 |  | 1.37 (1.14-1.64) | < 0.001 |
| Married | 1 |  |  | 1 |  |  | 1 |  |
| Divorced/separated/widowed | 1.47 (1.21-1.79) | < 0.001 |  | 1.40 (1.00-2.01) | 0.07 |  | 1.48 (1.24-1.76) | < 0.001 |
| Food security status |  |  |  |  |  |  |  |  |
| High | 1 |  |  | 1 |  |  | 1 |  |
| Marginal | 1.27 (1.05-1.54) | 0.01 |  | 1.84 (1.14-2.96) | 0.01 |  | 1.34 (1.12-1.60) | 0.001 |
| Low | 1.65 (1.31-2.08) | < 0.001 |  | 2.24 (1.36-3.68) | < 0.01 |  | 1.75 (1.42-2.16) | < 0.001 |
| Very low | 3.92 (2.66-5.78) | < 0.001 |  | 3.80 (1.44 -10.03) | 0.01 |  | 3.74 (2.62-5.33) | < 0.001 |
| Education |  |  |  |  |  |  |  |  |
| ≤ elementary school | 1.90 (1.52-2.34) | < 0.001 |  | 0.92 (0.54-1.56) | 0.75 |  | 1.71 (1.40-2.10) | < 0.001 |
| Middle school | 2.11 (1.64-2.72) | < 0.001 |  | 0.79 (0.43-1.44) | 0.43 |  | 1.82 (1.45-2.30) | < 0.001 |
| High school | 1.33 (1.10-1.61) | < 0.01 |  | 0.94 (0.59-1.50) | 0.81 |  | 1.27 (1.07-1.51) | 0.01 |
| ≥ College | 1 |  |  | 1 |  |  | 1 |  |
| Household income |  |  |  |  |  |  |  |  |
| 1st quartile (lowest) | 1.21 (1.00-1.53) | 0.11 |  | 2.23 (1.22-4.06) | 0.01 |  | 1.33 (1.07-1.66) | 0.01 |
| 2nd quartile | 0.97 (0.79-1.20) | 0.78 |  | 1.02 (0.60-1.74) | 0.95 |  | 0.96 (0.79-1.17) | 0.69 |
| 3rd quartile | 0.98 (0.80-1.21) | 0.86 |  | 0.71 (0.41-1.21) | 0.21 |  | 0.93 (0.76-1.13) | 0.45 |
| 4th quartile (highest) | 1 |  |  | 1 |  |  | 1 |  |
| Smoking |  |  |  |  |  |  |  |  |
| current | 1.54 (1.19-2.00) | 0.001 |  | 2.91 (1.65-5.11) | 0.001 |  | 1.72 (1.36-2.17) | < 0.001 |
| past | 1.43 (1.14-1.81) | 0.002 |  | 0.97 (0.55-1.70) | 0.91 |  | 1.38 (1.12-1.71) | < 0.01 |
| none | 1 |  |  |  | 1 |  | 1 |  |
| Alcohol intake |  |  |  |  |  |  |  |  |
| Heavy | 1.06 (0.85-1.33) | 0.60 |  | 0.88 (0.48-1.61) | 0.67 |  | 1.03 (0.84-1.27) | 0.76 |
| Moderate | 0.89 (0.76-1.03) | 0.12 |  | 0.74 (0.52-1.04) | 0.09 |  | 0.86 (0.75-0.99) | 0.03 |
| None | 1 |  |  | 1 |  |  | 1 |  |
