# Supplementary material for: Impact of sex and marital status on the prevalence of perceived depression in association with food insecurity
Source: PLoS One. 2020 Jun 11;15(6):e0234105. doi: 10.1371/journal.pone.0234105 (PMC7289387; doi:10.1371/journal.pone.0234105)
Supplement: S2 Table — (DOC) [file pone.0234105.s002.doc]

**Supplementary Table 2. Prevalence of perceived depression in association with sex-marital staus and food security status.^a^**

|  | High food security | Marginal food security | Low food security | Very low food security | Total | P value |
| --- | --- | --- | --- | --- | --- | --- |
| **2012, 2013, and 2015** |  |  |  |  |  |  |
| Male |  |  |  |  |  |  |
| never married (n=1,005) | 10.1 (1.1) | 2.9 (1.4) | 19.4 (4.7) | 23.0 (10.1) | 9.9 (1.0) | < 0.001 |
| married (n=4,418) | 6.3 (0.5) | 6.3 (1.1) | 9.3 (2.0) | 15.0 (7.1) | 6.5 (0.4) | 0.1 |
| divorced/widowed/separated (n=370) | 15.7 (3.0) | 19.8 (6.4) | 25.0 (6.8) | 75.7 (10.5) | 21.5 (2.6) | < 0.001 |
| Subtotal (n=5,793) | 7.7 (0.5) | 5.8 (0.9) | 13.9 (1.9) | 36.4 (6.9) | 8.2 (0.5) | < 0.001 |
| Female |  |  |  |  |  |  |
| never married (n=1,042) | 14.6 (1.3) | 15.2 (3.6) | 23.0 (5.9) | 18.8 (10.7) | 15.3 (1.2) | 0.3 |
| married (n=5,794) | 11.5 (0.6) | 23.7 (2.0) | 21.5 (2.4) | 40.3 (8.4) | 13.7 (0.6) | < 0.001 |
| divorced/widowed/separated (n=1,709) | 18.5 (1.4) | 24.7 (3.2) | 35.9 (4.2) | 45.2 (7.3) | 23.5 (1.3) | < 0.001 |
| Subtotal (n=8,545) | 13.0 (0.5) | 22.6 (1.6) | 26.4 (2.3) | 39.0 (5.5) | 15.6 (0.5) | < 0.001 |
| **2014** |  |  |  |  |  |  |
| Male |  |  |  |  |  |  |
| never married (n=312) | 4.9 (1.5) | 7.3 (4.7) | 18.0 (9.0) | 28.2 (24.2) | 6.3 (1.5) | 0.06 |
| married (n=1,418) | 3.1 (0.6) | 4.3 (1.9) | 3.2 (2.2) | 3.0 (3.1) | 3.2 (0.5) | 0.88 |
| divorced/widowed/separated (n=113) | 2.2 (1.1) | 2.6 (2.7) | 16.8 (10.7) | 29.8 (11.2) | 7.3 (2.2) | 0.001 |
|  |  |  |  |  |  |  |
| Subtotal (n=1,843) | 3.5 (0.6) | 5.1 (1.8) | 10.0 (4.4) | 8.2 (6.8) | 4.1 (0.6) | 0.07 |
| Female |  |  |  |  |  |  |
| never married (n=324) | 7.1 (1.9) | 26.2 (7.6) | 16.9 (7.7) | 15.0 (10.5) | 10.0 (1.9) | 0.001 |
| married (n=1,924) | 4.6 (0.6) | 12.3 (2.9) | 17.5 (4.5) | 47.1 (13.8) | 6.4 (0.7) | < 0.001 |
| divorced/widowed/separated (n=517) | 8.7 (1.7) | 15.0 (3.6) | 23.1 (7.3) | 57.3 (11.9) | 14.4 (1.7) | < 0.001 |
| Subtotal (n=2,765) | 4.6 (0.6) | 12.3 (2.9) | 17.5 (4.5) | 47.1 (13.8) | 6.4 (0.7) | < 0.001 |
| **Total (2012-2015)** |  |  |  |  |  |  |
| Male |  |  |  |  |  |  |
| never married (n=1,495) | 8.8 (1.0) | 3.9 (1.5) | 19.1 (4.2) | 24.3 (9.7) | 9.0 (0.8) | < 0.001 |
| married (n=6,313) | 5.5 (0.4) | 5.8 (1.0) | 8.1 (1.6) | 11.3 (5.2) | 5.7 (0.4) | 0.13 |
| divorced/widowed/separated (n=497) | 12.8 (2.4) | 15.6 (5.0) | 22.8 (5.8) | 53.2 (10.9) | 17.6 (2.1) | < 0.001 |
| Subtotal (n=8,305) | 6.7 (0.4) | 5.6 (0.8) | 13.1 (1.8) | 28.4 (5.6) | 7.2 (0.4) | <0.001 |
| Female |  |  |  |  |  |  |
| never married (n=1,478) | 12.8 (1.1) | 17.8 (3.3) | 21.7 (4.9) | 17.4 (7.9) | 14.1 (1.0) | 0.07 |
| married (n=7,835) | 9.7 (0.5) | 20.9 (1.7) | 20.6 (2.1) | 42.0 (7.2) | 11.8 (0.5) | < 0.001 |
| divorced/widowed/separated (n=2,248) | 16.3 (1.2) | 22.4 (2.6) | 33.3 (3.8) | 48.7 (6.4) | 21.4 (1.1) | < 0.001 |
| Subtotal (n=11,561) | 11.1 (0.4) | 20.8 (1.3) | 24.8 (1.9) | 40.5 (4.9) | 13.7 (0.4) | <0.001 |

^a^Data are presented as weighted percentage (standard error).
