# Supplementary material for: Impact of sex and marital status on the prevalence of perceived depression in association with food insecurity
Source: PLoS One. 2020 Jun 11;15(6):e0234105. doi: 10.1371/journal.pone.0234105 (PMC7289387; doi:10.1371/journal.pone.0234105)
Supplement: Attachment — Submitted filename: Response to reviewers.doc [file pone.0234105.s003.doc]

# Response letter

Dear Dr. Kotozaki,

Thank you for reviewing our manuscript PONE-D-19-21715 entitled “mpact of sex and marital status on the prevalence of perceived depression in association with food insecurity”. We appreciate your in-depth review and helpful comments. We have made corrections and modifications accordingly. The following are itemized lists stating our disposition in detail of each point raised by the reviewer. Thank you for your consideration of our manuscript. We look forward to a favorable decision on this revision.

Sincerely yours,

Yookyung Kim, Ph D

Professor

Department of Human Ecology, Graduate School, Korea University

**#1 reviewer:**

**Reviewer comment:** This study is of interest and is mostly technically sound. The only concern is that the PHQ-9, a respectable screener for depressive symptoms, was only used in one year (2014). The other years used a yes/no response regarding symptoms for at least 14 days over the last year. At face value this is not totally unreasonable approach, but this reviewer would be like to know if the prevalence estimates for the PHQ-9 year (2014) were comparable to the other years (2012, 2013, 2015). For this purpose it will be sufficient to estimate the overall prevalence of depression in year 2014 vs the other 3 years combined, with a t test. 95% CIs would be preferable for these estimates as a more informative epidemiological approach than standard errors re the precision of the point estimates.

***Response:*** *Thanks for the reviewer’s thoughtful comments. As the reviewer recommended, we compared the overall prevalence of depression in the year 2014 vs. the other three years combined. To see whether different usage of screening tool has influenced our conclusion, we also separately performed multiple regression analysis for perceived depression in the year 2014 vs. the other three years. A t-test showed that the point estimate of the prevalence of perceived depression in the year 2014 was lower than that of 3 years combined (5.6% vs. 11.9%, P < 0.001). The difference in point estimates was 5.4% (95% confidence interval: 4.2-6.7%). Multiple regression analysis showed similar results regardless of the year/period. That is, female sex and dissolution of marriage had a significant impact prevalence of perceived depression and synergistically contributed to a higher prevalence of perceived depression with food insecurity. We described these findings in the text (page 7, line 119-122; page 8, line 133-134. line 140-141; page 10, line 176-180 ) and newly prepared Supplementary Tables 1 & 2.*

**Supplementary Table 1. Adjusted odds ratio and 95% confidence interval for perceived depression.**

|  | **2012, 2013, and 2015** |  |  | **2014** |  |  | **Total (2012-2015)** |  |
| --- | --- | --- | --- | --- | --- | --- | --- | --- |
|  | Adjusted odds ratio  (95% confidence interval) | *P* value |  | Adjusted odds ratio  (95% confidence interval) | *P* value |  | Adjusted odds ratio  (95% confidence interval) | *P* value |
| Sex |  |  |  |  |  |  |  |  |
| Male | 1 |  |  | 1 |  |  | 1 |  |
| Female | 2.31 (1.86-2.88) | < 0.001 |  | 2.79 (1.71-4.56) | < 0.001 |  | 2.39 (2.00-2.92) | < 0.001 |
| Marital status |  |  |  |  |  |  |  |  |
| Never married | 1.33 (1.09-1.63) | 0.01 |  | 1.59 (1.02-2.47) | 0.04 |  | 1.37 (1.14-1.64) | < 0.001 |
| Married | 1 |  |  | 1 |  |  | 1 |  |
| Divorced/separated/widowed | 1.47 (1.21-1.79) | < 0.001 |  | 1.40 (1.00-2.01) | 0.07 |  | 1.48 (1.24-1.76) | < 0.001 |
| **….**  **….** | | | | | | | | |

**Supplementary Table 2. Prevalence of perceived depression in association with sex-marital staus and food security status.^a^**

|  | High food security | Marginal food security | Low food security | Very low food security | Total | P value |
| --- | --- | --- | --- | --- | --- | --- |
| **2012, 2013, and 2015** |  |  |  |  |  |  |
| Male |  |  |  |  |  |  |
| never married (n=1,005) | 10.1 (1.1) | 2.9 (1.4) | 19.4 (4.7) | 23.0 (10.1) | 9.9 (1.0) | < 0.001 |
| married (n=4,418) | 6.3 (0.5) | 6.3 (1.1) | 9.3 (2.0) | 15.0 (7.1) | 6.5 (0.4) | 0.1 |
| divorced/widowed/separated (n=370) | 15.7 (3.0) | 19.8 (6.4) | 25.0 (6.8) | 75.7 (10.5) | 21.5 (2.6) | < 0.001 |
| Subtotal (n=5,793) | 7.7 (0.5) | 5.8 (0.9) | 13.9 (1.9) | 36.4 (6.9) | 8.2 (0.5) | < 0.001 |
| Female |  |  |  |  |  |  |
| never married (n=1,042) | 14.6 (1.3) | 15.2 (3.6) | 23.0 (5.9) | 18.8 (10.7) | 15.3 (1.2) | 0.3 |
| married (n=5,794) | 11.5 (0.6) | 23.7 (2.0) | 21.5 (2.4) | 40.3 (8.4) | 13.7 (0.6) | < 0.001 |
| divorced/widowed/separated (n=1,709) | 18.5 (1.4) | 24.7 (3.2) | 35.9 (4.2) | 45.2 (7.3) | 23.5 (1.3) | < 0.001 |
| Subtotal (n=8,545) | 13.0 (0.5) | 22.6 (1.6) | 26.4 (2.3) | 39.0 (5.5) | 15.6 (0.5) | < 0.001 |
| **2014** |  |  |  |  |  |  |
| Male |  |  |  |  |  |  |
| never married (n=312) | 4.9 (1.5) | 7.3 (4.7) | 18.0 (9.0) | 28.2 (24.2) | 6.3 (1.5) | 0.06 |
| married (n=1,418) | 3.1 (0.6) | 4.3 (1.9) | 3.2 (2.2) | 3.0 (3.1) | 3.2 (0.5) | 0.88 |
| divorced/widowed/separated (n=113) | 2.2 (1.1) | 2.6 (2.7) | 16.8 (10.7) | 29.8 (11.2) | 5.3 (2.1) | 0.001 |
| Subtotal (n=1,843) | 3.5 (0.6) | 5.1 (1.8) | 10.0 (4.4) | 8.2 (6.8) | 4.1 (0.6) | 0.07 |
| Female |  |  |  |  |  |  |
| never married (n=324) | 7.1 (1.9) | 26.2 (7.6) | 16.9 (7.7) | 15.0 (10.5) | 10.0 (1.9) | 0.001 |
| married (n=1,924) | 4.6 (0.6) | 12.3 (2.9) | 17.5 (4.5) | 47.1 (13.8) | 6.4 (0.7) | < 0.001 |
| divorced/widowed/separated (n=517) | 8.7 (1.7) | 15.0 (3.6) | 23.1 (7.3) | 57.3 (11.9) | 14.4 (1.7) | < 0.001 |
| Subtotal (n=2,765) | 4.6 (0.6) | 12.3 (2.9) | 17.5 (4.5) | 47.1 (13.8) | 6.4 (0.7) | < 0.001 |
| **Total (2012-2015)** |  |  |  |  |  |  |
| Male |  |  |  |  |  |  |
| never married (n=1,495) | 8.8 (1.0) | 3.9 (1.5) | 19.1 (4.2) | 24.3 (9.7) | 9.0 (0.8) | < 0.001 |
| married (n=6,313) | 5.5 (0.4) | 5.8 (1.0) | 8.1 (1.6) | 11.3 (5.2) | 5.7 (0.4) | 0.13 |
| divorced/widowed/separated (n=497) | 12.8 (2.4) | 15.6 (5.0) | 22.8 (5.8) | 53.2 (10.9) | 17.6 (2.1) | < 0.001 |
| Subtotal (n=8,305) | 6.7 (0.4) | 5.6 (0.8) | 13.1 (1.8) | 28.4 (5.6) | 7.2 (0.4) | <0.001 |
| Female |  |  |  |  |  |  |
| never married (n=1,478) | 12.8 (1.1) | 17.8 (3.3) | 21.7 (4.9) | 17.4 (7.9) | 14.1 (1.0) | 0.07 |
| married (n=7,835) | 9.7 (0.5) | 20.9 (1.7) | 20.6 (2.1) | 42.0 (7.2) | 11.8 (0.5) | < 0.001 |
| divorced/widowed/separated (n=2,248) | 16.3 (1.2) | 22.4 (2.6) | 33.3 (3.8) | 48.7 (6.4) | 21.4 (1.1) | < 0.001 |
| Subtotal (n=11,561) | 11.1 (0.4) | 20.8 (1.3) | 24.8 (1.9) | 40.5 (4.9) | 13.7 (0.4) | <0.001 |

**Reviewer comments:** Re Table 1. BMI is not mentioned in the text unless I missed it. Model of Table 2: suggest dropping non-significant variables (age, BMI and physical activity). This will modify the other ORs a little, and that they are non-significant can be mentioned in the text.

***Response:*** *As the reviewer recommended, we deleted non-significant variables in Table 2. We mentioned it in the text (page 8, line 132-134). We revised the ORs in Table 2 (page 15:* Adjusted odds ratio*) and the text (page 8, line 127-131).*

**Reviewer comment:** Line 165: I do not understand the sentence beginning with “An alarmingly high…..”
***Response:*** *We deleted the sentence in the revised manuscript (page 8, line 168-169).*
